# Homer3 Promotes Aggressive Phenotypes in Triple-Negative Breast Cancer Through Cell Cycle- and MYC-Associated Programs

**DOI:** 10.3390/ijms27157009

**Published:** 2026-08-04

**Authors:** Kuei-Yen Tsai, Yu-Jia Chang, Jang-Chun Lin, G. M. Shazzad Hossain Prince, Uyanga Batzorig, Ai-Wei Lee, Chin-Sheng Hung

**Affiliations:** 1 Graduate Institute of Clinical Medicine, College of Medicine, Taipei Medical University, Taipei 11031, Taiwan; leiftsai@gmail.com (K.-Y.T.); r5424012@tmu.edu.tw (Y.-J.C.); k2220707@gmail.com (J.-C.L.); princegmsh@tmu.edu.tw (G.M.S.H.P.); 2 Department of Surgery, School of Medicine, College of Medicine, Taipei Medical University, Taipei 11031, Taiwan; 3 Division of General Surgery, Department of Surgery, Shuang Ho Hospital, Taipei Medical University, New Taipei City 23561, Taiwan; 4 Cancer Research Center and Translational Laboratory, Department of Medical Research, Taipei Medical University Hospital, Taipei Medical University, Taipei 11031, Taiwan; 5 Cell Physiology and Molecular Image Research Center, Wan Fang Hospital, Taipei Medical University, Taipei 11696, Taiwan; 6 Department of Pathology, Wan Fang Hospital, Taipei Medical University, Taipei 11696, Taiwan; 7 Department of Radiology, School of Medicine, College of Medicine, Taipei Medical University, Taipei 11031, Taiwan; 8 Department of Dermatology, University of California, La Jolla, San Diego, CA 92093, USA; buyangaaa@yahoo.com; 9 Department of Anatomy and Cell Biology, School of Medicine, College of Medicine, Taipei Medical University, Taipei 11031, Taiwan

**Keywords:** breast cancer, TNBC, Homer family, Homer3, MYC, cell cycle

## Abstract

Homer proteins (Homer1–3) are scaffold proteins that mediate protein–protein interactions in signal transduction; however, the role of Homer3 in breast cancer (BC), particularly triple-negative breast cancer (TNBC), remains poorly defined. Here, we investigated the clinical relevance and functional significance of Homer3 in BC and TNBC. Publicly available datasets from TCGA and GEO were analyzed to evaluate associations between Homer3 expression and patient outcomes using Kaplan–Meier survival analysis. Pathway enrichment analysis and gene set variation analysis (GSVA) were performed to identify signaling programs associated with Homer3 co-expressed genes. Functional roles were examined using stable TNBC cell lines with Homer3 knockdown or overexpression, followed by assays for cell proliferation, clonogenic growth, migration, invasion, and wound healing. We found that Homer3 expression was elevated in breast tumors compared with normal tissues and was associated with poor prognosis in both BC and TNBC. Homer3 expression was higher in TNBC than in non-TNBC subtypes and negatively correlated with estrogen receptor (ER) and progesterone receptor (PR) expression. Functionally, Homer3 depletion suppressed TNBC cell proliferation, clonogenic capacity, migration, invasion, and wound healing, whereas Homer3 overexpression produced reciprocal effects. Pathway analyses revealed that Homer3 co-expressed genes were enriched in cell cycle-related pathways and Hallmark MYC signaling, which were associated with adverse clinical outcomes. Consistently, Homer3 knockdown selectively reduced key proliferative cell-cycle regulators. Collectively, these findings demonstrate that Homer3 is associated with aggressive phenotypes and MYC- and cell cycle-linked proliferative programs in breast cancer, particularly in TNBC. Survival analyses are presented as exploratory findings, supporting the biological relevance of Homer3 in TNBC. These findings suggest that Homer3 may represent a potential therapeutic vulnerability.

## 1. Introduction

Breast cancer (BC) is the most common cancer-related cause of death for women [1]. In 2012, there were 1.67 million new instances of BC diagnosed, making up 25% of all female malignancies globally [2]. The five-year survival rate in industrialized nations is 80%; in poor nations, it is only 40% [3]. BC incidence was estimated to increase to 85 per 100,000 women by 2021 [4]. In the United States, one in eight women is diagnosed with BC, whereas in Asia, the prevalence is lower, with one in 35 women affected. BC is more prevalent in densely populated parts of South Asian developing nations [5,6]. Males can also develop BC, in addition to females [7]. Numerous diagnostic techniques are employed, including digital mammography, MRI, breast biopsy, fine-needle aspiration, core biopsy, nuclear medicine, single photon emission computed tomography, and positron emission tomography. Several biomarkers have been used to predict disease progression in BC patients, such as CA15-3, CA2.29, and HER2 [8,9]. However, the specificity and sensitivity of these markers remain suboptimal, necessitating the identification of more reliable prognostic and diagnostic biomarkers.

BC is classified into several categories based on histological characteristics and invasiveness. Stage 0 is considered non-invasive, whereas stage 4 represents the most invasive form of the disease [10]. TNBC is an aggressive subtype that accounts for approximately 15% of all BC cases. It is characterized by the absence of estrogen receptor (ER) and progesterone receptor (PR) expression, along with low or excessive expression of HER2 (also known as ERBB2) [11]. Generally, treatment includes lumpectomy, mastectomy, ovarian ablation, anti-estrogen therapy, aromatase inhibitors (AIs), radiation therapy, and chemotherapy, as well as targeted molecular therapies [12]. For hormone-responsive BC, selective estrogen receptor modulators (SERMs), selective estrogen receptor downregulators (SERDs), and (AIs) are used for therapy [13,14]. The defining biological characteristics of TNBC include high proliferative capacity, extensive immune cell infiltration, basal-like and mesenchymal phenotypes, androgen receptor (AR) expression, and loss of BRCA1 function [15]. These aggressive characteristics make TNBC more refractory to available endocrine or anti-HER2 therapy [15,16]. There are relevant chemotherapies for TNBCs, and several clinical trials have been conducted to compare the effects of chemotherapeutic agents, including anthracycline–taxane, carboplatin, or bevacizumab to neoadjuvant chemotherapy, cisplatin plus gemcitabine, iniparib, capecitabine, neoadjuvant epirubicin, and cyclophosphamide followed by docetaxel, with or without carboplatin [17]. MiR-205 and miR-342 have been proposed as potential biomarkers for TNBC diagnosis [18]. Several molecular targets, including androgen receptor, epidermal growth factor receptor (EGFR), poly-adenosine diphosphate ribose polymerase (PARP), and vascular endothelial growth factor (VEGF), are considered promising therapeutic targets for TNBC [19]. PARP plays an important role in DNA damage repair, and its related pathway using specific inhibitors such as iniparib, olaparib, and veliparib shows potential in enhancing tumor cell responses to chemotherapy and radiotherapy.

Additionally, targeting HER2 tyrosine kinase by their specific inhibitors, such as gefitinib, erlotinib, lapatinib, and trastuzumab, constitutes one of the most effective strategies of targeted therapeutic approaches [20]. Trastuzumab, a humanized monoclonal antibody that binds to a specific epitope of the HER2/neu (c-erbB-2) protein, not only reduces malignancy but also enhances tumor cell sensitivity to endocrine therapy and specific chemotherapeutic agents [21]. Lapatinib inhibits the dual epidermal growth factor receptor and HER2 tyrosine kinase [22]. A previous study involving 12,858 BC patients reported an increased risk of lung and brain metastases in HER2-positive cases [23]. Inhibition of the early signaling pathway is essential for metastasis-targeted therapy, and inhibition of epithelial–mesenchymal transition (EMT) leads to the prevention of early steps of metastasis [24]. A clinical trial demonstrated that switching from paclitaxel to cisplatin enhanced progression-free survival in the metastatic situation in advanced TNBC, which was not chosen. The U.S. Food and Drug Administration (FDA) has approved olaparib, a PARP inhibitor, for HER2-negative metastatic BC in BRCA mutant patients [25]. Ribociclib and abemaciclib are inhibitors of cyclin-dependent kinases 4 and 6 (CDK4/6), being used in combination with an AI for postmenopausal patients with HR-positive, HER2-negative advanced, or metastatic BC as initial endocrine therapy [26,27,28]. However, the molecular mechanisms of BC still need to be further dissected to obtain promising drugs for the advanced and metastatic stages of BC.

The Homer protein family is known as a cytoplasmic scaffolding protein and is divided into short and long Homer proteins based on their structure [29]. Homer-1A, Homer-2C, Homer-2D, Homer-3C, and Homer-3D are considered as short Homers whereas Homer-1B, Homer-1C, Homer-2A, Homer-2B, Homer-3A, and Homer-3B are long Homers [29]. All Homer proteins comprise a conserved N-terminal Ena/Vasp homology one domain, which have been found to bind to a variety of proteins, including the proline-rich sequence found in group 1 metabotropic glutamate receptors (mGluRs), inositol-1,4,5-triphosphate receptors (IP_3_Rs), and transient receptor potential canonical-1 (TRPC1) ion channels regulating Ca^2+^ homeostasis [30,31,32].

Homer3 is highly expressed in the brain, skeletal muscle, and heart. Its functions have mainly been studied in neurons, and it is localized to excitatory synapses and modulates calcium signaling, axon guidance, and dendritic spine remodeling, and is also responsive to the slow phase of the action potential [29,30,33]. It has also been reported that the dissociation of the amyloid precursor protein (APP)/Homer3 complex is regulated by Ca^2+^ intracellular concentration [34]. Homer3 modulates transcription and is essential in differentiating muscle and the nervous system [35]. Previous studies have reported that Homer3 is overexpressed in acute myeloid leukemia (AML), while decreased expression of Homer3 is associated with poor prognosis in AML [35,36]. It has also been demonstrated that the forced expression of Homer3 in myelogenous leukemia cell lines, such as K562 cells, inhibits proliferation, influences the cell cycle, and affects apoptosis induced by As_2_O_3_ by downregulating Bcl-2 expression in leukemia [35]. Homer3 interacts with WBP2, a protein known to promote tumor progression in glioma [37]. Recently, Homer3 has been found to have an upregulated gene in estrogen-resistant BC and esophageal cancer, although its precise role remains unclear [38,39]. In addition, Homer3 facilitates c-Src-mediated β-catenin tyrosine phosphorylation via epidermal growth factor receptor (EGFR) activation and leads to breast cancer progression [40]. These findings suggest that Homer3 may serve as a novel therapeutic target for BC.

## 2. Results

### 2.1. Homer3 Is Associated with Breast Cancer Progression

**Figure 1:**
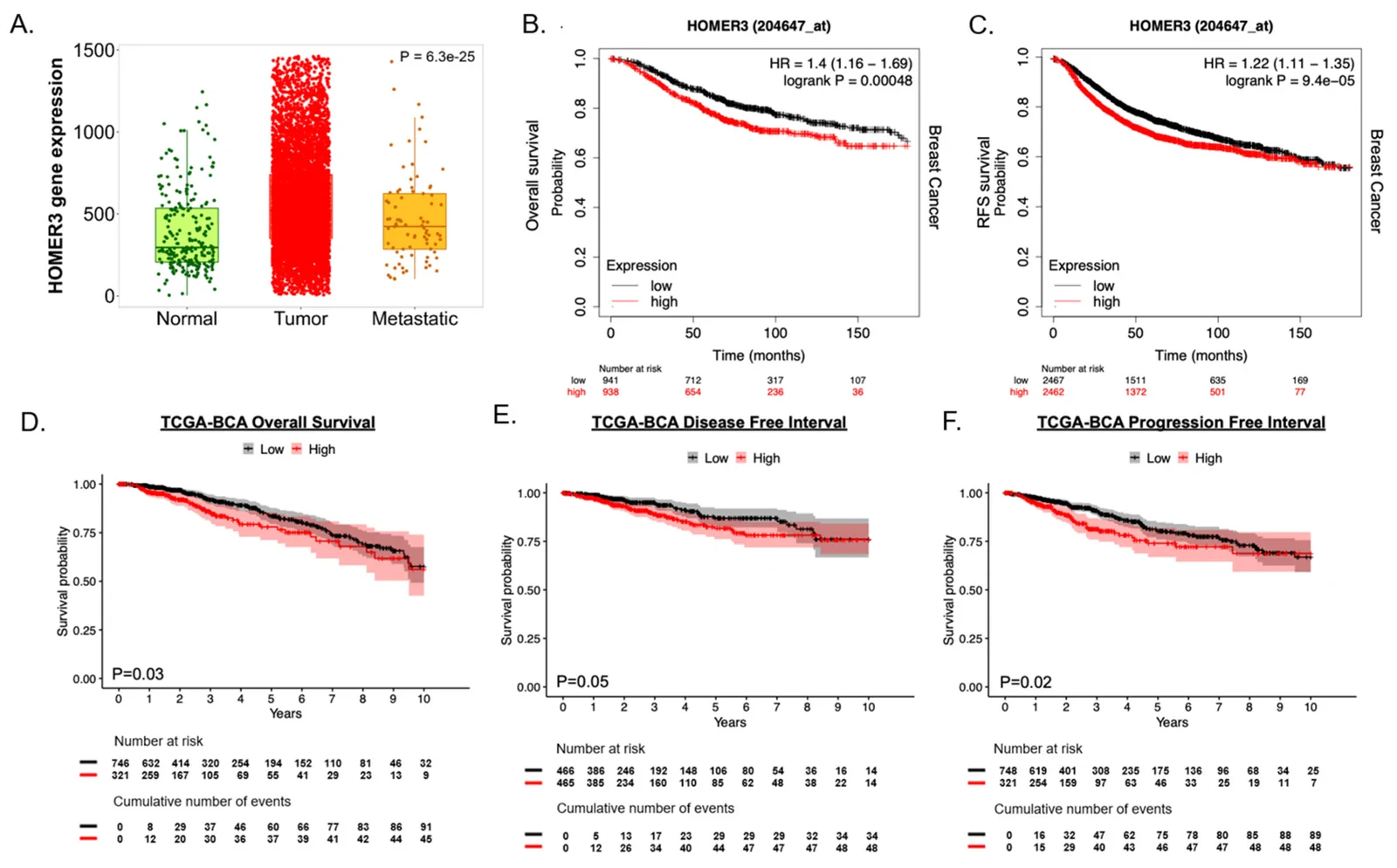
**Homer3 expression is elevated in breast cancer and correlates with poor clinical outcomes.** (**A**) Differential expression analysis of Homer3 in normal breast tissues and breast cancer samples based on publicly available datasets. (**B**,**C**) Kaplan–Meier plots of overall survival (*n* = 1880) and relapse-free survival (*n* = 4934) of breast cancer patients based on the gene-chip expression levels of Homer3. (**D**–**F**) Survival probability curves for overall survival, disease-free interval, and progression-free interval of breast cancer patients (*n* = 1069) according to Homer3 expression levels, based on TCGA datasets. Statistical significance was calculated using the KM log-rank *p*-value. HR: Hazard ratio calculated using the Cox-proportional hazards model. (**A**) Gene expression analysis using the TNMplot database; (**B**,**C**) GEO datasets using the KMplotter platform; (**D**–**F**) TCGA-BRCA dataset using R-based tools.

We investigated the role of Homer3 in breast adenocarcinoma (BC) progression. As shown in Figure 1A, we found that Homer3 is highly expressed (Kruskal–Wallis *p*-value < 0.001) in both primary tumors (*n* = 7569) and metastatic tumors (*n* = 82) compared to normal tissues (*n* = 242). We further analyzed different patient cohorts to assess the survival probability of BC patients based on high versus low Homer3 expression. In both the GEO dataset (*n* = 1880; HR: 1.4, *p* < 0.001) and TCGA-BRCA dataset (*n* = 1069; HR: 1.5, *p* < 0.05), high Homer3 expression was associated with a poorer overall survival rate in patients (Figure 1B,D). Worse relapse-free survival (GEO dataset, *n* = 4934) was associated with a higher median expression of Homer3, as shown in Figure 1C. Similarly, as shown in Figure 1E,F, BC patients with high Homer3 expression from the TCGA-BRCA dataset (*n* = 1069) exhibited a shorter disease-free interval (HR: 1.5, *p* < 0.05) and progression-free interval (HR: 1.5, *p* < 0.05). Furthermore, Cox proportional hazards analyses were performed using the available clinicopathological variables in the TCGA-BRCA cohort to further evaluate the prognostic significance of Homer3 (Table S1). In the univariable analysis, Homer3 expression was not significantly associated with overall survival (HR = 1.209, 95% CI: 0.879–1.663, *p* = 0.244). In contrast, age and pathologic stage remained independent prognostic factors after multivariable adjustment. These findings suggest that although Homer3 expression is associated with adverse clinical outcomes in Kaplan–Meier analyses, its independent prognostic value could not be established in the overall TCGA-BRCA cohort.

To address the potential influence of tumor purity, we analyzed the correlation between Homer3 expression and tumor purity estimates obtained from TIMER3.0 (Figure S1). Homer3 showed only weak correlations with tumor purity in the overall BRCA cohort (r = −0.065) and molecular subtypes, including basal-like (r = 0.126), HER2-enriched (r = 0.057), LumA (r = −0.061), and LumB tumors (r = −0.127). These results suggest that tumor purity is unlikely to be a major determinant of Homer3 expression. Taken together, these survival analyses provide exploratory evidence supporting the biological relevance of Homer3 in TNBC. However, these findings should be interpreted cautiously because potential confounding by molecular subtype and other clinicopathological variables cannot be completely excluded.

### 2.2. Homer3 Expression Is Negatively Correlated with Hormone Receptor Status in Breast Cancer

**Figure 2:**
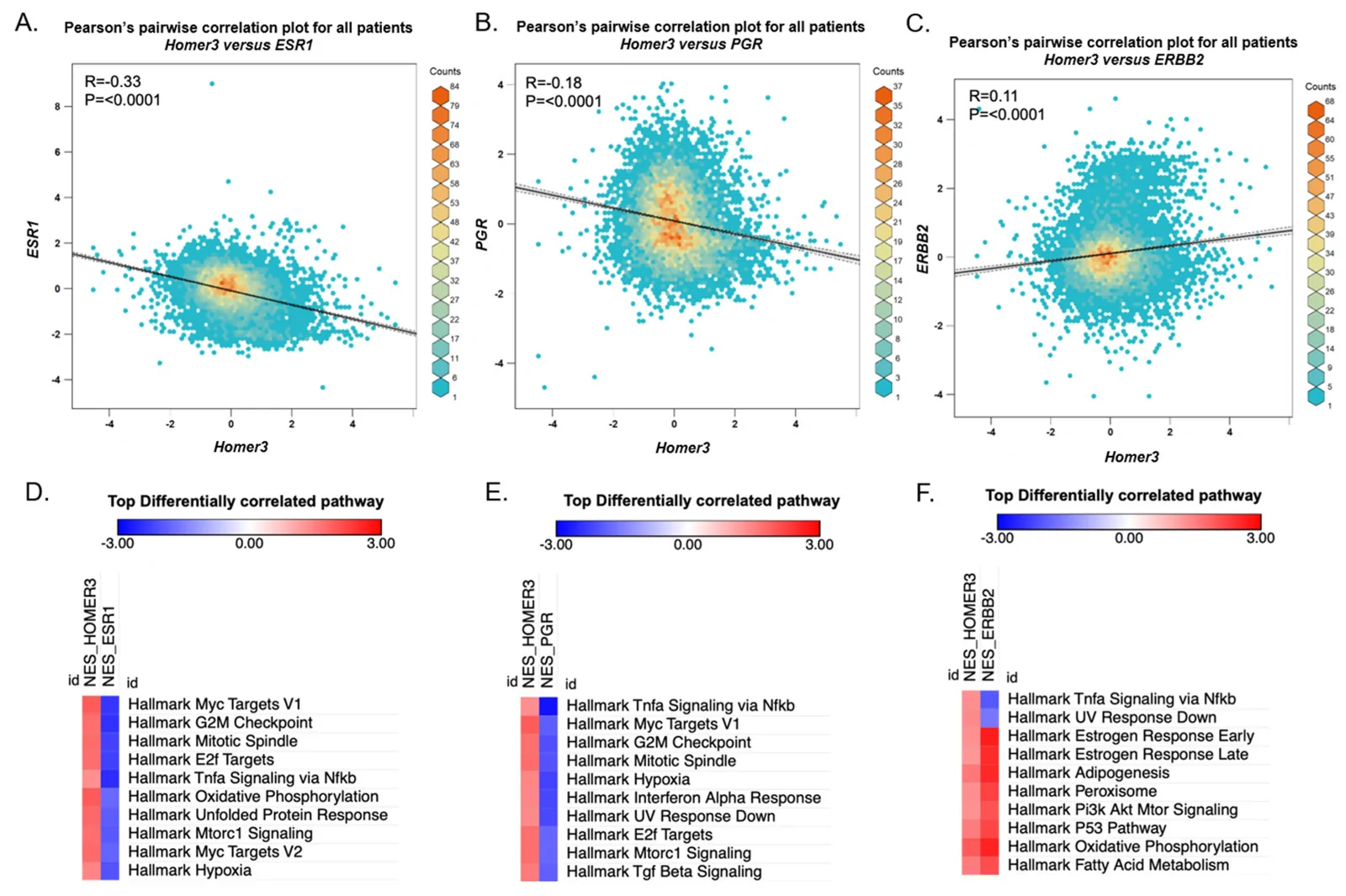
**Homer3 negatively correlates with ER and PR in BRCA.** (**A**–**C**) Pearson’s correlation analysis was performed on gene expression data from breast cancer patients (*n* = 10,872). (**D**–**F**) Heatmap displaying the NES of the top differentially enriched Hallmark pathways, based on single-gene co-expression analysis in breast cancer patients. Red indicates positive enrichment, whereas blue indicates negative enrichment. ESR1: estrogen receptor (ER); PGR: progesterone receptor (PR); ERBB2: human epidermal growth factor receptor 2. NES: normalized enrichment score. Figure 2: correlation analyses specifically for breast cancer gene expression databases using the bc-GenExMiner v5.0 platform.

BC is driven by hormone receptors, such as estrogen receptor (ER), progesterone receptor (PR), and the growth factor receptor HER2 (ERBB2). Therefore, we analyzed the co-expression patterns of Homer3 with these receptors in BC patients (*n* = 10,872). Surprisingly, we found that Homer3 negatively correlates with ER (r = −0.33, *p* < 0.0001) and PR (r = −0.18, *p* < 0.0001) but positively correlates with the HER2 receptor (r = 0.11, *p* < 0.0001) (Figure 2A–C). We performed gene set enrichment analysis (GSEA) to investigate these correlations further and identify the top differentially correlated Hallmark pathways (Figure 2D–F). We generated a heatmap to visualize and compare the top differentially enriched pathways based on the variance in normalized enrichment score (NES). Our analysis revealed that most Hallmark pathways (e.g., Hallmark MYC targets V1, Hallmark G2M checkpoint, Hallmark mitotic spindle, and Hallmark E2F targets) that were positively enriched in the Homer3-high group were downregulated in the ER- and PR-high group (Figure 2D,E). However, the top enriched Hallmark pathways in Homer3 and HER2 exhibited a positive correlation (Figure 2F).

### 2.3. Homer3 Supports Breast Cancer Cell Survival and Correlates with Adverse Outcome in Triple-Negative Breast Cancer

**Figure 3:**
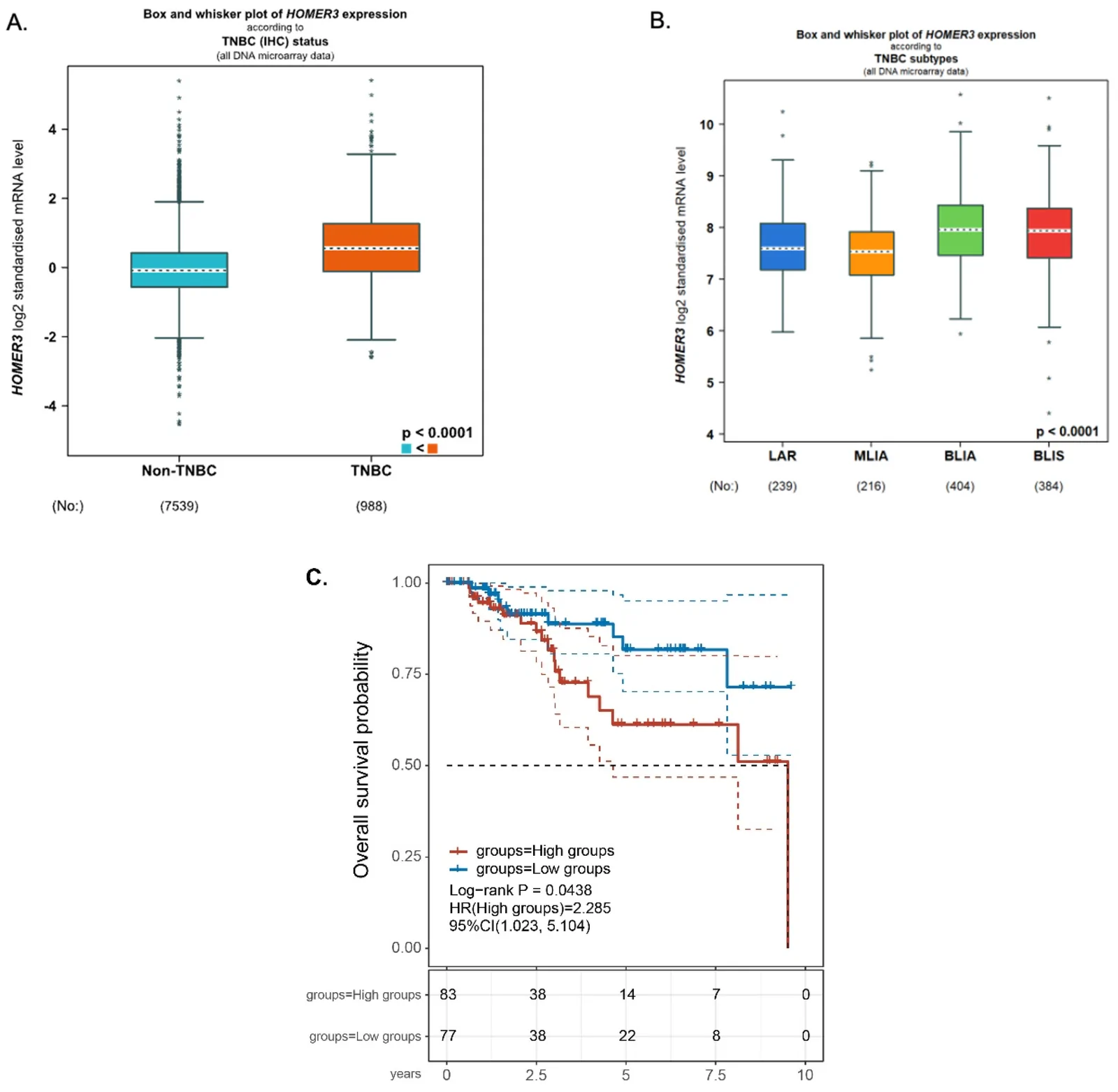
**Homer3 determines poor outcome in TNBC patients.** (**A**) Homer3 expression level in non-TNBC and TNBC patients based on DNA microarray data (*n* = 8527). (**B**) Homer3 expression levels in TNBC subtypes. (**C**) The Kaplan–Meier (KM) survival curve of the Homer3 gene in TNBC (based on TCGA data), with inter-group differences assessed using the log-rank test. HR (High exp) denotes the hazard ratio of the high-expression group compared to the low-expression group. The 95% confidence interval (CI) indicates the range of uncertainty for the HR estimate. BLIA: basal-like immune-activated; BLIS: basal-like immune-suppressed; LAR: luminal androgen receptor; MLIA: mesenchymal-like immune-activated; TNBC: triple-negative breast cancer. (**A**,**B**) DNA microarray pooled data using bc-GenExMiner v5.0; * represents outliers in the datasets. (**C**) TCGA-BRCA TNBC subset using bc-GenExMiner survival analysis.

**Figure 4:**
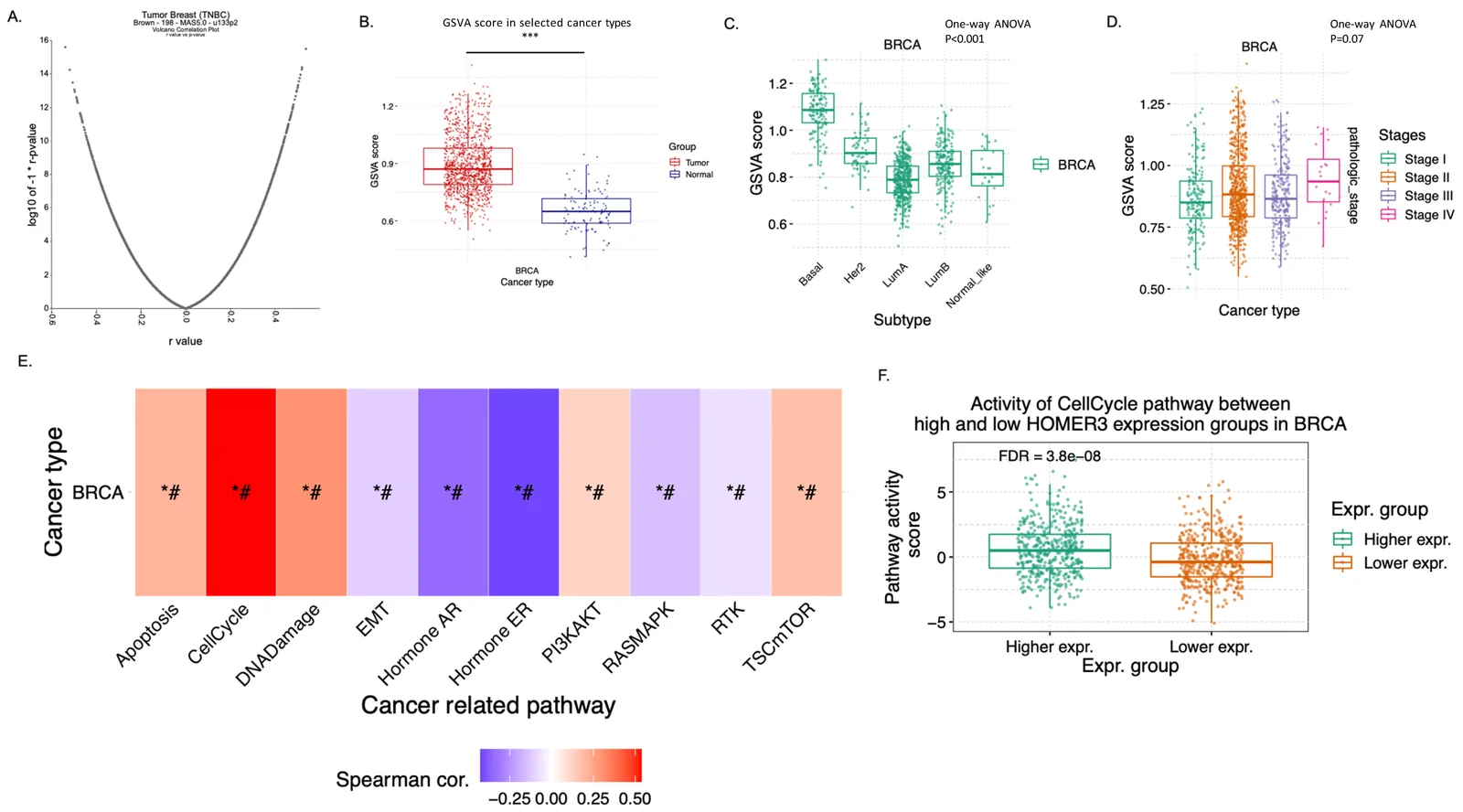
**Top Homer3 co-expressed genes regulate cell survival gene sets in breast cancer.** (**A**) Volcano plot depicting the correlation of significantly positive and negatively co-expressed genes with Homer3 in TNBC (GSE76124). (**B**) GSVA score of the top 500 positively co-expressed Homer3 genes in BC. *** *p* ≤ 0.001. (**C**) GSVA score of the top 500 positively co-expressed Homer3 genes across BC subtypes. (**D**) GSVA score of the top 500 positively co-expressed Homer3 genes across different BC stages. (**E**) Heatmap illustrates the correlation between the GSVA score of the top 500 positively co-expressed Homer3 genes and enriched cancer-related pathway. * *p* ≤ 0.05, # FDR ≤ 0.05. (**F**) Activity of the cell cycle pathway in BC, comparing high versus low Homer3 expression groups. (**A**) correlation analysis of GSE76124 dataset using bc-GenExMiner; (**B**–**F**) TCGA-BRCA dataset using the Gene Set Cancer Analysis (GSCA) platform with gene set variation analysis (GSVA) algorithm analysis.

Although Homer3 expression is linked to poor survival outcomes in BC patients (Figure 1), we found that Homer3 negatively correlates with hormone receptors in BC (Figure 2). Therefore, we investigated Homer3 expression levels in TNBC. As shown in Figure 3A, Homer3 expression was significantly higher in the TNBC patients (*n* = 988) compared to non-TNBC patients (*n* = 7539). Among the TNBC subtypes, the basal-like immune-activated (BLIA) and basal-like immune-suppressed (BLIS) subgroups exhibited higher Homer3 expression (Figure 3B). Next, we retrieved the TNBC gene expression data and clinical information from TCGA to perform a Kaplan–Meier survival analysis. As shown in Figure 3C, TNBC patients with high Homer3 expression levels had a trend toward poorer overall survival in exploratory analysis (HR = 2.285, *p* < 0.05), though this result should be interpreted with caution given the limited sample size and absence of multivariate adjustment. Since Homer3 negatively correlates with hormone receptors (Figure 2), we aimed to investigate whether Homer3 promotes breast cancer and TNBC progression by regulating other key molecular pathways. As shown in Figure 4A, we plotted genes significantly co-expressed with Homer3 in TNBC (GSE76124, *n* = 198). Then, we selected the top 500 most significantly positively co-expressed genes to perform gene set variation analysis (GSVA) in BC [41]. The GSVA score of Homer3 co-expressed genes was higher in tumors than in normal tissues (Figure 4B). Next, we calculated the GSVA score of this gene set across different pathological BRCA stages. We found that basal-like and HER2-positive tumors exhibited significantly higher GSVA scores (Figure 4C). Similarly, the GSVA score progressively increased in higher BC grades, with stage IV tumors displaying a significantly higher score than stage I tumors (Figure 4D). We conducted pathway enrichment analysis to explore further the involvement of cancer-related pathways in the Homer3 co-expressed gene set. As shown in Figure 4E, the cell cycle pathway was the most significantly correlated (FDR < 0.001). Other pathways regulating cell survival, such as the PI3K/AKT and mTOR pathways, were also positively correlated. In contrast, hormone-related pathways (AR and ER) were negatively correlated, consistent with our findings in Figure 2. Finally, we confirmed that high Homer3 expression activates the cell cycle pathway in BC, further supporting its role in regulating cell cycle progression in BC (Figure 4F).

### 2.4. Homer3 Is Associated with TNBC Cell Proliferation

**Figure 5:**
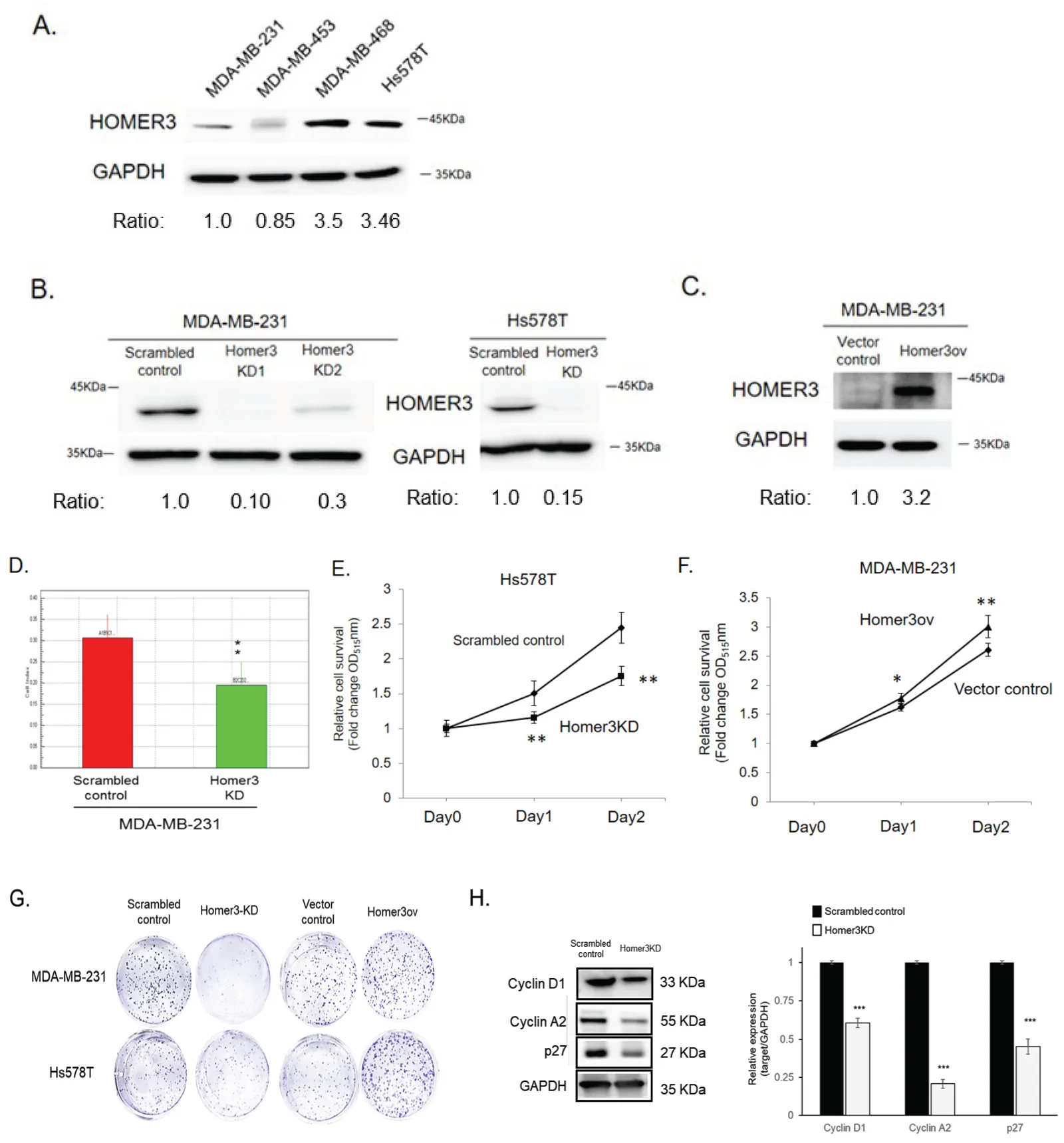
**Homer3 regulates cell proliferation and selective cell-cycle programs in triple-negative breast cancer.** (**A**) Western blot analysis showing endogenous Homer3 expression in TNBC cell lines, with GAPDH as a loading control. (**B**) Validation of stable Homer3 knockdown in MDA-MB-231 and Hs578T cells by Western blot. (**C**) Validation of Homer3 overexpression in MDA-MB-231 cells compared with the vector control. Panels B and C were derived from independent Western blot experiments performed to validate Homer3 knockdown and overexpression, respectively. Densitometric analyses were performed independently for each experiment, with Homer3 expression normalized to GAPDH and expressed relative to the corresponding control group (set to 1.0). Accordingly, comparisons should be interpreted only within each experiment. (**D**) Real-time cell proliferation analysis of MDA-MB-231 cells following Homer3 knockdown, measured using the xCELLigence biosensor system. (**E**) Cell proliferation assay of Hs578T cells following Homer3 knockdown. (**F**) Cell proliferation assay of MDA-MB-231 cells following Homer3 overexpression. The raw absorbance (OD515) values for all SRB assay experiments are provided in Figure S2. (**G**) Colony formation assay demonstrating that Homer3 knockdown reduces clonogenic capacity, whereas Homer3 overexpression enhances colony-forming ability in TNBC cells. (**H**) Western blot analysis showing reduced expression of Cyclin D1 and Cyclin A2, accompanied by altered p27 levels following Homer3 knockdown. Protein expression was quantified by densitometric analysis using ImageJ, normalized to GAPDH, and expressed relative to the corresponding control group (set to 1.0), supporting a selective regulatory effect on proliferative cyclins rather than a generalized cell-cycle arrest. * *p* < 0.05; ** *p* < 0.01; *** *p* < 0.001.

We discovered that Homer3 and its co-expressed gene set are primarily associated with cell cycle- and cell survival-related pathways. To further investigate the functional role of Homer3 in TNBC, we examined whether Homer3 modulates cell proliferation. As shown in Figure 5A, Homer3 expression levels were analyzed across the TNBC cell lines. We then established two stable Homer3 knockdown cell lines (MDA-MB-231 and Hs578T) and one Homer3-overexpressing cell line (MDA-MB-231), as validated in Figure 5B,C. Functional assays revealed that Homer3 knockdown significantly suppressed cell proliferation in both MDA-MB-231 and Hs578T cells (Figure 5D,E). In contrast, Homer3 overexpression markedly enhanced the proliferation rate of MDA-MB-231 cells (Figure 5F). Consistent with these findings, colony formation assays demonstrated that Homer3 depletion significantly reduced clonogenic capacity, whereas Homer3 overexpression increased colony-forming ability (Figure 5G). To provide molecular evidence supporting the observed proliferative effects, we further analyzed representative cell cycle-related mediators. As shown in Figure 5H, Homer3 knockdown resulted in a pronounced reduction in Cyclin D1 and Cyclin A2 expression, accompanied by altered p27 levels. Collectively, these findings indicate that Homer3 depletion selectively attenuates proliferative cell-cycle progression, consistent with the reduced short- and long-term proliferative phenotypes observed in TNBC cells.

### 2.5. Homer3 Is Associated with Metastatic Phenotypes in Triple-Negative Breast Cancer

**Figure 6:**
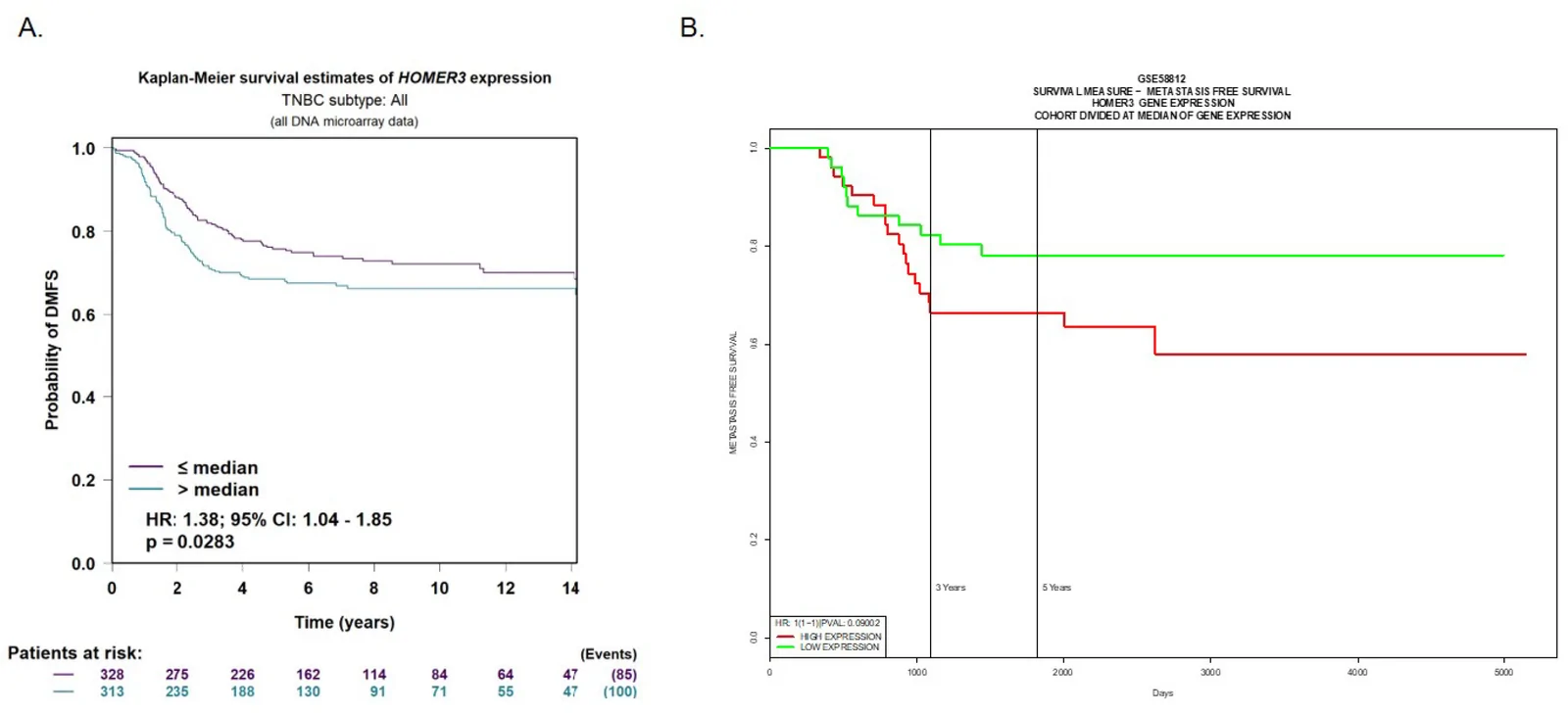

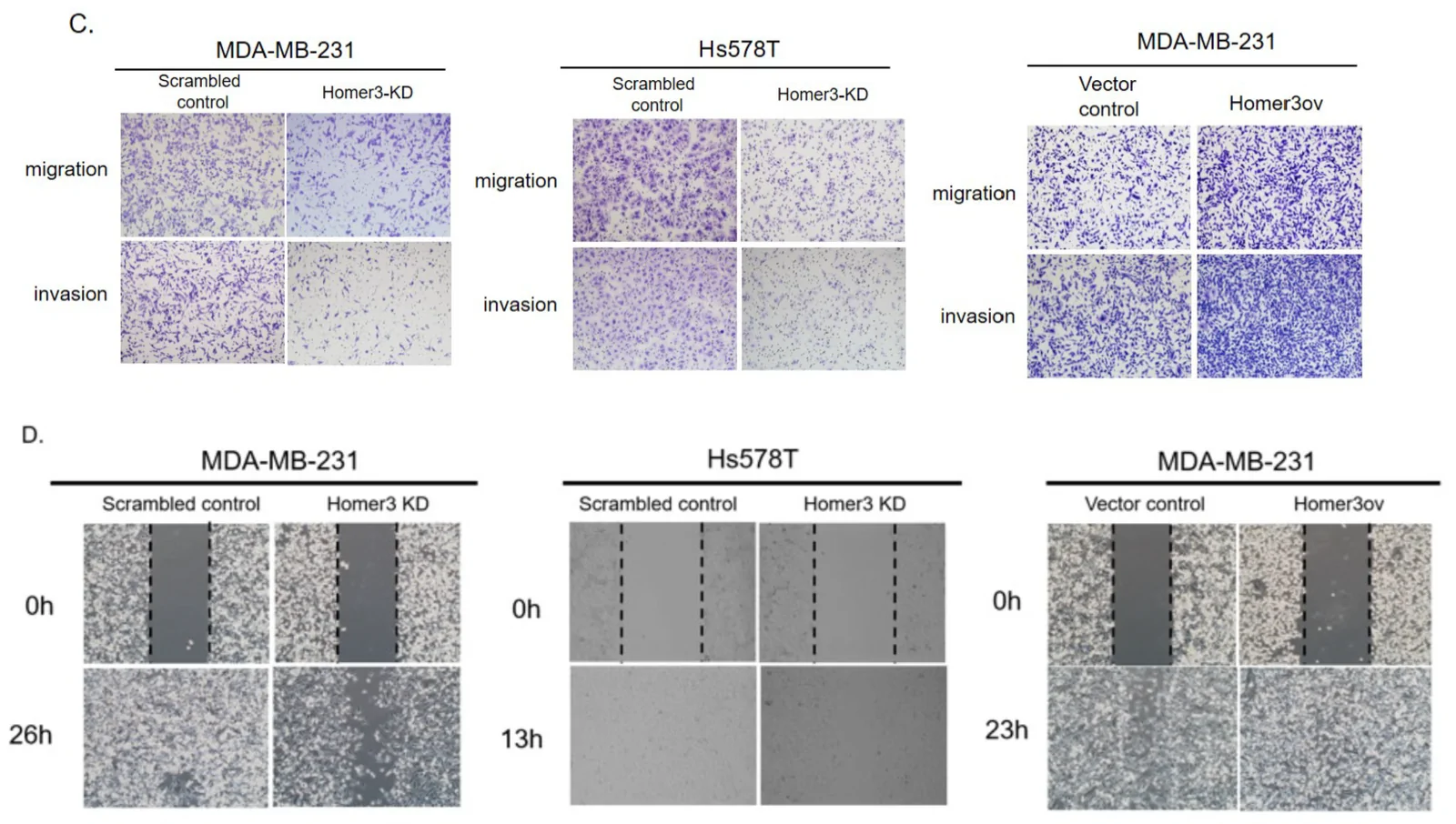
**Homer3 regulates metastatic properties in triple-negative breast cancer.** (**A**,**B**) Distant metastasis-free survival analysis of TNBC patients stratified by Homer3 expression levels. (**C**) Transwell migration and invasion assays showing the effects of Homer3 knockdown in MDA-MB-231 and Hs578T cells, and Homer3 overexpression in MDA-MB-231 cells. (**D**) Wound healing assay demonstrating the effects of Homer3 knockdown in MDA-MB-231 and Hs578T cells, and Homer3 overexpression in MDA-MB-231 cells. (**A**) GEO datasets using KMplotter platform; (**B**) GSE58812 dataset using PrognoScan platform.

Since Homer3 regulates cell proliferation in TNBC, we investigated its role in promoting TNBC metastasis. Using publicly available DNA microarray data, we plotted a survival curve based on the median cut-off of Homer3 expression levels. As shown in Figure 6A, high Homer3 expression (above the median) was significantly associated with poorer distant metastasis-free survival in TNBC patients (HR: 1.38, *p* < 0.05). In another TNBC patient cohort, high Homer3 expression consistently exhibited a similar trend toward worse metastasis-free survival, although the association did not reach statistical significance (HR: 1, *p* = 0.09002; Figure 6B). Our in vitro data are consistent with these findings (Figure 6C,D). In a transwell migration assay, we observed that Homer3 knockdown significantly reduced the migration rate, whereas Homer3 overexpression enhanced it (Figure 6C). Similarly, the transwell invasion assay revealed that TNBC cells exhibited a lower invasion rate upon Homer3 knockdown but a higher invasion rate upon Homer3 overexpression (Figure 6C). We also investigated the wound-healing ability of TNBC cells regarding Homer3 expression levels. As shown in Figure 6D, Homer3 silencing significantly reduced the wound healing rate, whereas Homer3 overexpression enhanced wound closure ability.

### 2.6. Homer3 Is Associated with Activation of Key Hallmark Pathways in Breast Cancer and Triple-Negative Breast Cancer

**Figure 7:**
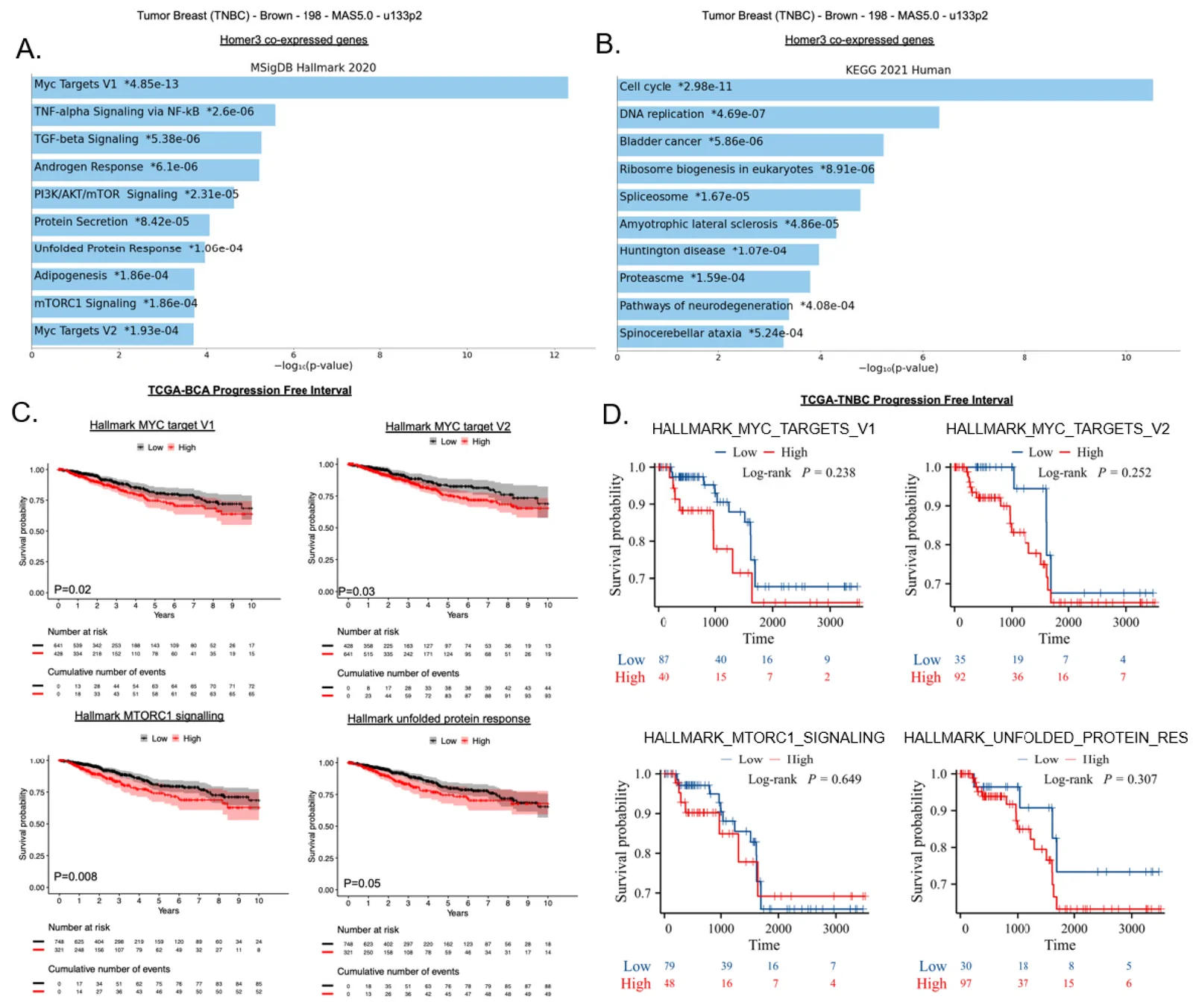

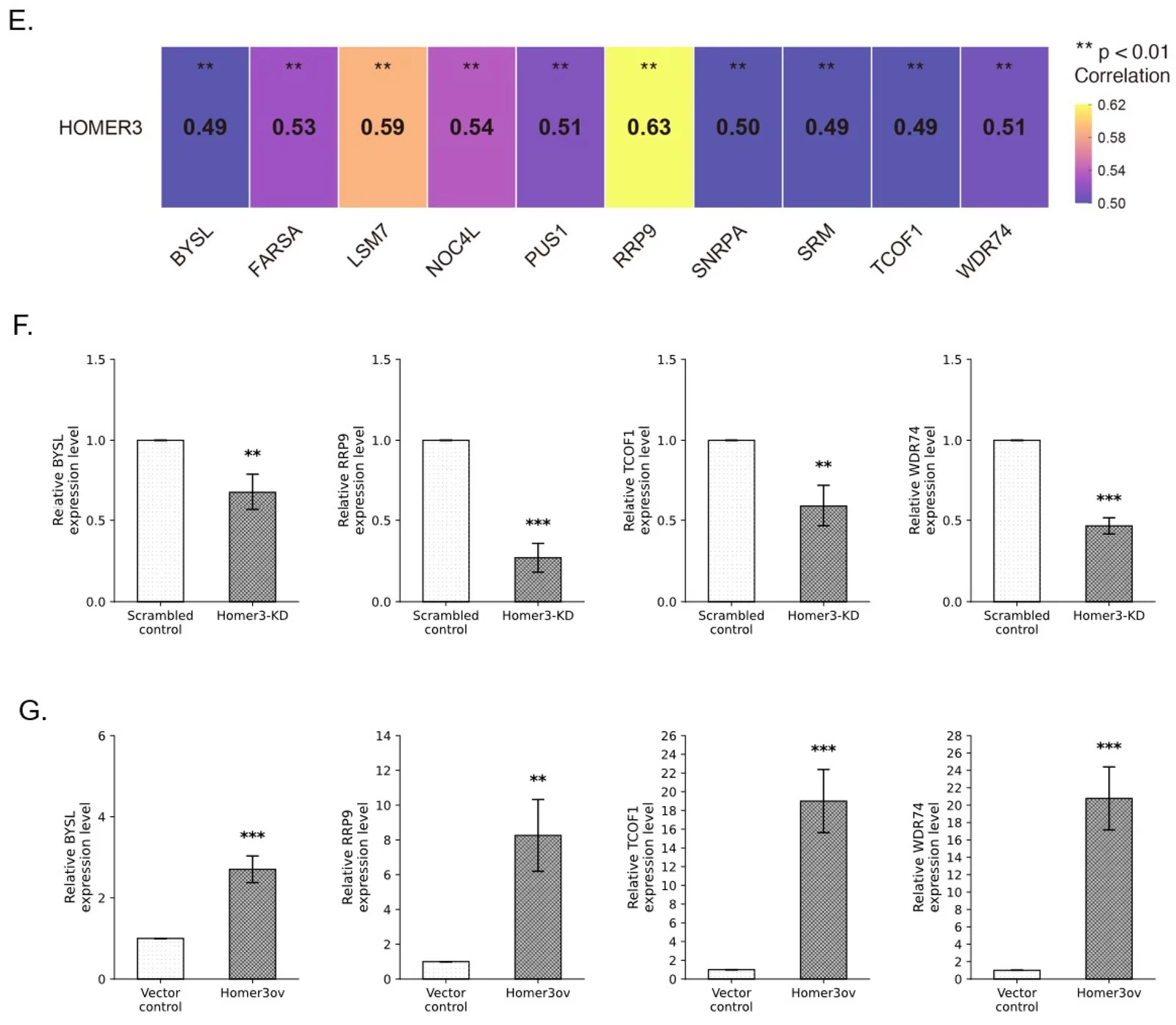
**Homer3-associated Hallmark gene sets correlate with breast cancer progression and MYC-associated transcriptional outputs.** (**A**,**B**) Hallmark and KEGG pathway enrichment analyses of genes positively co-expressed with Homer3 in triple-negative breast cancer (TNBC) samples from the GSE76124 dataset, highlighting enrichment of proliferation- and MYC-associated gene sets. * indicates the exact p-value for pathway enrichment (expressed in scientific notation). (**C**,**D**) Kaplan–Meier analyses of progression-free interval (PFI) demonstrating that enrichment of Homer3-associated Hallmark gene sets, including MYC-related pathways, is significantly associated with adverse clinical outcomes in breast cancer and TNBC cohorts. (**E**) Correlation heatmap of Homer3 and MYC-associated genes in TCGA-TNBC. (**F**) Quantitative reverse transcription-PCR (qRT-PCR) analysis showing reduced expression of representative MYC-associated genes following Homer3 knockdown in TNBC cells. (**G**) qRT-PCR analysis demonstrating reciprocal upregulation of these MYC-associated transcriptional outputs upon Homer3 overexpression. Data are presented as mean ± SEM from at least three independent experiments. qRT-PCR analyses were performed to provide experimental validation of representative MYC-associated transcriptional outputs identified by pathway enrichment analyses. (**A**,**B**) Gene set enrichment analysis based on GSE76124 using GSCA; (**C**,**D**) TCGA-BRCA, TCGA-TNBC, and Hallmark gene sets in the Molecular Signature Database using the GSCA platform; (**E**) TCGA-TNBC dataset using R-based tools. ** *p* < 0.01; *** *p* < 0.001.

To investigate the molecular programs underlying Homer3-mediated breast cancer and TNBC progression, we analyzed genes positively co-expressed with Homer3 in TNBC using the GSE76124 dataset. Pathway enrichment analyses revealed significant enrichment of Hallmark and KEGG gene sets associated with cell-cycle regulation and oncogenic signaling (Figure 7A,B). Consistently, KEGG pathway analysis identified cell cycle as one of the most significantly enriched pathways among Homer3 co-expressed genes in TNBC. Among the positively enriched Hallmark gene sets, upregulation of Hallmark MYC targets V1, Hallmark MYC targets V2, Hallmark MTORC1 signaling, and Hallmark unfolded protein response was significantly associated with poorer progression-free interval (PFI) in BC patients (*p* < 0.05), as demonstrated by Kaplan–Meier survival analyses (Figure 7C). Similar survival analyses were further performed in TNBC cohorts (Figure 7D). Although these associations did not reach statistical significance in TNBC, enrichment of Hallmark MYC targets V1 and V2 gene sets exhibited a consistent trend toward poorer PFI, suggesting a potential contribution of MYC-associated programs to TNBC progression. To address whether Homer3-associated MYC/cell-cycle signatures primarily reflect generalized proliferation, we examined the correlation between Homer3 and MKI67 expression, using MKI67 as a surrogate proliferation marker. In the overall BRCA cohort, Homer3 showed only a very weak correlation with MKI67 expression (r = 0.0708, *p* = 0.0189), whereas no significant correlation was observed in the TNBC subset (r = 0.0332, *p* = 0.677) (Figure S3). These suggest that Homer3 expression in TNBC is unlikely to simply represent MKI67-defined proliferative activity. To further validate the relevance of these pathways in TNBC, we analyzed two independent GEO datasets (GSE65194 and GSE45827) to identify gene sets enriched in TNBC and basal-like breast cancer. Differential expression analyses and pathway enrichment consistently demonstrated the enrichment of MYC-associated Hallmark gene sets and the KEGG cell cycle pathway in TNBC and basal-like subtypes (Figure S4), supporting the robustness of our findings. Based on the transcriptomic identification of enriched MYC-associated gene sets, we next examined whether Homer3 modulates MYC-associated transcriptional outputs. The correlation heatmap revealed that Homer3 showed a significant positive correlation with MYC-related genes (Figure 7E). Bidirectional perturbation of Homer3 revealed that knockdown reduced, whereas overexpression increased, the expression of representative MYC-associated genes, including BYSL, RRP9, TCOF1, and WDR74, as determined by qRT-PCR (Figure 7F,G, Figure S5). These results provide experimental validation supporting the association between Homer3 expression and the activation of MYC-linked transcriptional programs in TNBC cells.

## 3. Discussion

In this study, we demonstrate that Homer3 contributes to breast cancer progression and is associated with adverse survival outcomes in TNBC, despite subtype-specific differences in downstream gene regulation. Notably, Homer3 expression was inversely correlated with estrogen receptor (ER) and progesterone receptor (PR) expression. Several Hallmark pathways positively associated with Homer3—including MYC signaling, E2F targets, mitotic spindle, and MTORC1 signaling—were negatively associated with ER and PR expression. These findings suggest that Homer3 may represent a previously unrecognized regulator of hormone receptor–negative breast cancer subtypes, particularly TNBC.

Although higher Homer3 expression was associated with poorer survival in Kaplan–Meier analyses, Cox regression analyses using the available clinicopathological variables did not identify Homer3 as an independent prognostic factor in the overall TCGA-BRCA cohort. Therefore, these findings support the biological relevance of Homer3 but should not be interpreted as establishing it as an independent prognostic biomarker. Additional studies incorporating more comprehensive clinicopathological information and independent validation cohorts will be required to clarify the prognostic significance of Homer3 in breast cancer.

Cell growth and division are governed by a tightly regulated cell-cycle machinery, and disruption of this process is a fundamental driver of uncontrolled proliferation in cancer. In TNBC, tumor progression is sustained by the activation of multiple oncogenic signaling programs despite the absence of ER, PR, and HER2, highlighting the need to identify actionable regulatory nodes [42,43,44]. Our data indicate that Homer3 depletion reduced the expression of Cyclin A2 and Cyclin D1, which are essential for S-phase progression and G1/S transition, respectively. In addition, altered p27 expression suggests that loss of Homer3 disrupts cell-cycle regulatory balance in a context-dependent manner rather than through a single CDK inhibitory pathway. This pattern supports a model in which Homer3 promotes proliferative capacity through the selective regulation of key cyclins rather than the induction of a generalized cell-cycle process.

The selective downregulation of proliferative cyclins upon Homer3 knockdown is consistent with our transcriptomic analyses demonstrating the suppression of MYC-associated transcriptional programs. MYC is a central regulator of genes involved in cell-cycle progression and biosynthetic processes, and MYC-driven outputs frequently manifest as the coordinated regulation of specific cyclins rather than uniform changes across all cell-cycle components. Dysregulation of MYC signaling is well-documented in breast cancer, particularly in TNBC, where elevated MYC activity is associated with aggressive tumor behavior and therapeutic resistance [45,46]. Consistent with this, our pathway analyses identified significant enrichment of MYC-associated gene sets among Homer3-correlated transcripts, and higher MYC pathway enrichment was associated with adverse clinical outcomes in both BC and TNBC cohorts.

Previous studies have reported that Homer3 can facilitate growth factor-mediated β-catenin signaling [40], and activation of the Wnt/β-catenin pathway is known to induce MYC expression through TCF/LEF-dependent transcription [47]. Based on these observations, we propose that Homer3 may function upstream to modulate signaling networks converging on MYC-associated transcriptional programs. Consistent with this possibility, we found that several MYC-associated genes previously implicated in ribosome biogenesis and cellular growth, including BYSL, RRP9, TCOF1, and WDR74, were positively associated with Homer3 expression and were consistently regulated following Homer3 knockdown and overexpression. These findings provide experimental support for the transcriptomic analyses while remaining consistent with an indirect regulatory model. It is noteworthy that NOC4L exhibited discordant expression patterns between the TCGA co-expression analysis and our qRT-PCR validation (Figure S5). Specifically, NOC4L showed a positive correlation with Homer3 in the TCGA-TNBC cohort but displayed the opposite response following Homer3 perturbation in isogenic cell models. This discrepancy may reflect differences between population-level co-expression analyses and acute transcriptional responses following experimental manipulation, and therefore NOC4L was excluded from the primary validation panel. Taken together, these findings support an association between Homer3 and MYC-associated transcriptional outputs rather than direct activation of MYC signaling. Further studies will be required to define the molecular intermediates linking Homer3 to these transcriptional programs.

Recent studies have implicated Homer3 in cancer progression and identified it as a potential biomarker in several malignancies, including bladder cancer [48]. Given the aggressive biology and marked heterogeneity of TNBC [49], identifying biomarkers that are applicable across breast cancer subtypes remains an important clinical objective [50].

From a therapeutic perspective, the lack of druggable hormone receptors or HER2 amplification allows TNBC refractory to the endocrine and anti-HER2 therapies, highlighting an urgent need to identify alternative treatment targets. The scaffold function of Homer3, which mediates protein–protein interactions at postsynaptic densities and in growth factor signaling complexes, suggests that its oncogenic activity may be amenable to disruption by small-molecule inhibitors targeting the EVH1 domain or by strategies that interfere with its interaction with downstream effectors such as β-catenin [40]. It is worth noting that Homer3-mediated facilitation of c-Src–dependent β-catenin tyrosine phosphorylation downstream of EGFR activation places Homer3 at a nodal point. Furthermore, Tong et al. demonstrated that Homer3 preferentially engages the CC domain to recruit Src kinase and displace LATS1-mediated YAP1 phosphorylation, thereby sustaining high YAP1 nuclear activity and driving tumor growth across multiple cancer contexts [51]. Homer3 may integrate receptor tyrosine kinase signaling with nuclear transcriptional outputs, including MYC, suggesting that Homer3 may represent a potential therapeutic vulnerability worthy of further investigation. In addition, the elevated Homer3 expression observed in basal-like and HER2-enriched breast cancer subtypes, as well as its increase across tumor stages, indicates that Homer3 inhibition may exhibit preferential activity in high-risk patient populations. While direct pharmacological targeting of Homer3 remains to be developed, the present study provides a rationale for future research and drug discovery.

Several limitations should be acknowledged. These results were largely derived from cell-based experiments and the examination of publicly available clinical data, which inherently limits their ability to represent the full intricacies of the in vivo tumor environment. Whether the Homer3–MYC pathway holds genuine translational value remains unconfirmed, as it has not been tested in xenograft or patient-derived xenograft systems. Future work using such animal models will be necessary to establish its clinical applicability.

Collectively, our findings support Homer3 as a biologically relevant regulator of breast cancer progression, particularly in TNBC, through the modulation of MYC-associated proliferative programs and multiple oncogenic signaling pathways. These findings provide mechanistic insights into the role of Homer3 in hormone receptor–negative breast cancer and support its potential as a therapeutic target for future investigation.

## 4. Materials and Methods

### 4.1. Cell Culture

TNBC cell lines (MDA-MB-231, MDA-MB-453, MDA-MB-468, and Hs578T) were obtained from the American Type Culture Collection (ATCC, Manassas, VA, USA). Cells were cultured in DMEM (Invitrogen, Carlsbad, CA, USA) supplemented with 10% heat-inactivated FBS, 100 U/mL penicillin, and 100 μg/mL streptomycin (Life Technologies, Carlsbad, CA, USA) and maintained at 37 °C in a humidified incubator with 5% CO_2_.

### 4.2. Generation of Homer3 Knockdown and Overexpression TNBC Cell Lines

Homer3 overexpression was achieved using a human Homer3 expression plasmid (RC204168) obtained from OriGene Technologies, Inc. (Rockville, MD, USA). Homer3 knockdown was performed using two specific short hairpin RNAs (shRNAs): TRCN0000153533 (CGGCTAAAGAAGATGTTGTCT) and TRCN0000155724 (CAAACCAAGGACCAGGAGATT), purchased from the RNAi Core Facility, Taipei, Taiwan. Cells were transfected using the Neon^®^ Transfection System (Life Technologies, Thermo Fisher Scientific, Carlsbad, CA, USA) according to the manufacturer’s instructions [52]. Briefly, cells were washed twice with phosphate-buffered saline (PBS), and 1.5 × 10^5^ cells were resuspended in 9 µL of T-buffer mixed with 1 µL of plasmid DNA. Electroporation was performed using a single pulse at 1.4 kV for 20 ms with the pipette-style Neon microporator. Following transfection, cells were cultured in complete medium and selected with puromycin for approximately two weeks to establish stable knockdown or overexpression cell lines. The efficiency of Homer3 knockdown or overexpression was confirmed by quantitative PCR (qPCR) and Western blot analysis.

### 4.3. Cell Proliferation by xCELLigence Biosensor Real Time Monitor System

The cell proliferation assay was performed using an RTCA DP instrument (ACEA BioSciences, Inc. San Diego, CA, USA) in a 5% CO_2_ humidified incubator at 37 °C [53]. In brief, 5000 cells were planted on an E-plate 16 (ACEA Biosciences Inc.) in RPMI medium supplemented with FBS. For 4 h, the plate was monitored every 30 s and subsequently every 30 min. The data were examined using the RTCA software 2.0 associated with the instrument.

### 4.4. Cell Viability Assay-Sulforhodamine B (SRB) Assay

Cells were seeded in 96-well plates at a density of 5000 cells per well in RPMI medium containing 10% FBS and incubated overnight. Cells were treated with drugs at the indicated concentrations and incubated for 24, 48, or 72 h. Cell viability was assessed using the sulforhodamine B (SRB) assay as previously described [54]. Briefly, cells were fixed with 10% trichloroacetic acid at 4 °C, stained with 0.4% SRB in 1% acetic acid, and solubilized with 20 mM Tris buffer (pH 7.4). Absorbance was measured at 515 nm using a Bio-Rad microplate reader.

### 4.5. Colony Formation

Cells were seeded in 6-well plates at a density of 1000 cells per well and cultured under standard conditions (37 °C, 5% CO_2_) without medium change. After 10 days, colonies were fixed with 0.4% formaldehyde and stained with 0.1% crystal violet. Colonies containing more than 50 cells were counted and quantified using ImageJ software (version 1.53).

### 4.6. Quantitative Reverse-Transcription Polymerase Chain Reaction

Total RNA was extracted using TRIzol reagent (Invitrogen, Life Technologies, Thermo Fisher Scientific, Carlsbad, CA, USA) and reverse-transcribed from 1 µg RNA using a cDNA Synthesis Kit (Invitrogen) in a 20 µL reaction. Quantitative PCR was performed with SYBR Green Master Mix (HD Life Sciences, Boston, MA, USA) on an ABI 7500 FAST Real-Time PCR System. Reactions (20 µL) were run under the following conditions: 95 °C for 10 min, followed by 45 cycles of 95 °C for 15 s and 60 °C for 1 min. All samples were analyzed in technical triplicates across at least three independent biological replicates, and relative gene expression was normalized to GAPDH. Primer sequences were as follows: BYSL-F, 5′-CTCTCCAACTGGGAGCAAATCC-3′; BYSL-R, 5′-TGCGTTCCTTCAGGTTAGAGGC-3′; RRP9-F, 5′-GTTGGTGGCAAAAGAGATCCAGG-3′; RRP9-R, 5′-CTTTGGCAGCAGAGAAGATGGC-3′; TCOF1-F, 5′-CAGGAAGACTCTGAGAGCAGTG-3′; TCOF1-R, 5′-CCCTTTTGCTGAAGGTGCTCTG-3′; WDR74-F, 5′-GAAAACAGGCGGCGAACTTCAC-3′; WDR74-R, 5′-TGCTGAAGTGCTTCACCGTCCT-3′.

### 4.7. Migration Assay

A BD Falcon cell culture insert (BD Biosciences, San Jose, CA, USA) with a pore size of 8 µm was used for the migration test [55]. Cells (1 × 10^5^) suspended in serum-free medium were seeded into the upper chamber, and medium containing 10% FBS was added to the lower chamber. After 48 h, non-migrated cells were removed, while migrated cells were fixed with methanol, stained with 0.1% crystal violet, and counted under an Olympus IX71 inverted microscope at 100× magnification.

### 4.8. Invasion Assay

Cell invasive ability was determined using a 24-well BD BioCoat™ Matrigel Invasion Chamber (BD Biosciences) [56]. Cells (1 × 10^5^) in serum-free medium were seeded into Matrigel-coated inserts, with 10% FBS medium in the lower chamber. After 48 h, invaded cells were fixed, stained with 0.1% crystal violet, imaged, and quantified under an Olympus IX71 microscope at 100× magnification.

### 4.9. Wound-Healing Assay

The ibidi culture-inserts system performed the wound-healing migration assay [57]. Cells (3.5 × 10^5^) were seeded into inserts and incubated overnight. After insert removal, wound closure was monitored in fresh medium using time-lapse microscopy with a 4× objective (Lumascope 500X Etaluma, Inc., Carlsbad, CA, USA).

### 4.10. Protein Extraction and Western Blot Analysis

Protein expression was analyzed by SDS–PAGE followed by Western blotting [58]. Cells were lysed in lysis buffer (Sigma, St. Louis, MO, USA, C2978) supplemented with protease inhibitors (Complete Protease Inhibitor Tablets; Boehringer Mannheim). Equal amounts of protein (20 μg) were resolved on 4–20% EzPAGE precast gels and transferred using the iBlot 3 system (Thermo Fisher Scientific, Waltham, MA, USA). Membranes were blocked and incubated overnight at 4 °C with primary antibodies against Homer3 (Proteintech, Rosemont, IL, USA, 1624-1-AP), GAPDH, Cyclin A2, Cyclin D1 (iReal Biotechnology, Hsinchu City, Taiwan), and p27 (Santa Cruz Biotechnology, Dallas, TX, USA), followed by HRP-conjugated secondary antibodies (1:5000). Signals were detected using enhanced chemiluminescence (T-Pro Biotechnology, New Taipei City, Taiwan) and visualized with the ChemiDoc Imaging System (Bio-Rad, Hercules, CA, USA).

### 4.11. Bio-Informatic Data Resources

Publicly available datasets from TCGA and GEO were analyzed using R-based tools and publicly accessible platforms. STAR-count RNA-Seq data and corresponding clinical information for TNBC samples were obtained from TCGA, converted to TPM values, and log2(TPM + 1) normalized. A total of 160 TNBC cases with complete expression and clinical data were included for downstream analyses. Spearman correlation analysis was performed to evaluate the association between Homer3 expression and MYC target genes. The correlation heatmap was generated in R using the pheatmap package. Kaplan–Meier survival analyses were performed, and hazard ratios (HRs) with 95% confidence intervals (CIs) were calculated using log-rank tests and Cox proportional-hazards models implemented in the survival R package (v3.2.11) [59]. TNMplot and KMplotter were used for gene expression comparison and survival probability estimation [60,61]. Gene–gene correlation analyses were conducted using the bc-GenExMiner platform [62], and differentially correlated pathways were visualized using correlation analysis tools in R. Pathway enrichment analyses of Hallmark and KEGG gene sets were performed using EnrichR [63]. Homer3 co-expressed genes in TNBC were retrieved from the GSE76124 dataset and analyzed using the Gene Set Cancer Analysis (GSCA) server [64].

### 4.12. Statistical Analysis

All experiments were performed at least three times. Data are presented as mean ± SD. Statistical significance between two groups was determined using a two-tailed Student’s *t*-test. Asterisks indicate significant differences (*p* < 0.05), as specified in the figure legends.

## 5. Conclusions

TNBC is an aggressive subtype of breast cancer with limited therapeutic options, underscoring the need for an improved understanding of the molecular programs driving disease progression. In this study, we demonstrate that Homer3 expression is associated with aggressive phenotypes and MYC- and cell cycle-linked proliferative programs, with exploratory survival associations in breast cancer, particularly in TNBC. Functional and transcriptomic analyses reveal that Homer3 is linked to enhanced proliferative capacity and activation of cell cycle– and MYC-associated transcriptional programs, supporting its role in sustaining malignant progression. Although further mechanistic studies are required to define the precise signaling intermediates involved, our findings suggest that Homer3 represents a potential therapeutic vulnerability in both TNBC and non-TNBC breast cancer subtypes and may provide a foundation for the development of novel treatment strategies aimed at improving patient outcomes.

## Data Availability

The original contributions presented in this study are included in the article/Supplementary Materials. Further inquiries can be directed to the corresponding authors.

## Supplementary Materials

The following supporting information can be downloaded at: https://www.mdpi.com/article/10.3390/ijms27157009/s1.
