# Supplementary material for: Homer3 Promotes Aggressive Phenotypes in Triple-Negative Breast Cancer Through Cell Cycle- and MYC-Associated Programs
Source: Int J Mol Sci. 2026 Aug 4;27(15):7009. doi: 10.3390/ijms27157009 (PMC13467463; doi:10.3390/ijms27157009)
Supplement: Supplementary file 1 [file ijms-27-07009-s001.zip › revised_Figure S1.pdf]

Spearman's ρ : positive correlation (p<0.05, ρ>0)
Spearman's ρ : negative correlation (p<0.05, ρ<0
Spearman's ρ : not significant (p>0.05)

cancer	tumor purity ESTIMATE
BRCA (n=1086)	-0.065
BRCA-Basal (n=188)	0.126
BRCA-Her2 (n=82)	0.057
BRCA-LumA (n=560)	-0.061
BRCA-LumB (n=216)	-0.127

Figure S1. Correlation between Homer3 expression and tumor purity in breast cancer. TIMER3.0 analysis showing the correlation between Homer3 expression and ESTIMATE-derived tumor purity in the TCGA-BRCA cohort and molecular subtypes. Spearman's correlation coefficients are shown.
