# Supplementary material for: Homer3 Promotes Aggressive Phenotypes in Triple-Negative Breast Cancer Through Cell Cycle- and MYC-Associated Programs
Source: Int J Mol Sci. 2026 Aug 4;27(15):7009. doi: 10.3390/ijms27157009 (PMC13467463; doi:10.3390/ijms27157009)
Supplement: Supplementary file 1 [file ijms-27-07009-s001.zip › revised_Figure S2.pdf]

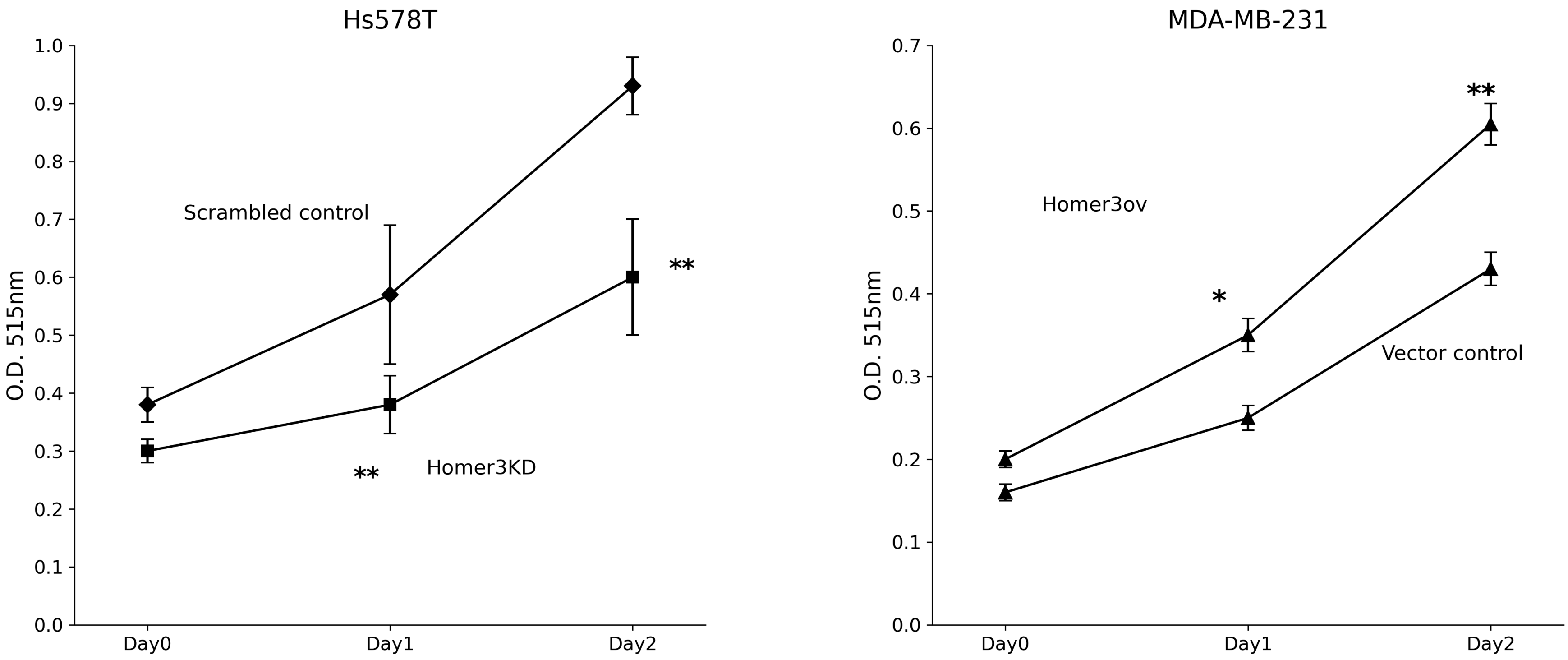

Figure S2. Cell proliferation assay of breast cancer cells following HOMER3 perturbation, presented as O.D. values.
