# Supplementary material for: Homer3 Promotes Aggressive Phenotypes in Triple-Negative Breast Cancer Through Cell Cycle- and MYC-Associated Programs
Source: Int J Mol Sci. 2026 Aug 4;27(15):7009. doi: 10.3390/ijms27157009 (PMC13467463; doi:10.3390/ijms27157009)
Supplement: Supplementary file 1 [file ijms-27-07009-s001.zip › revised_Figure S3.pdf]

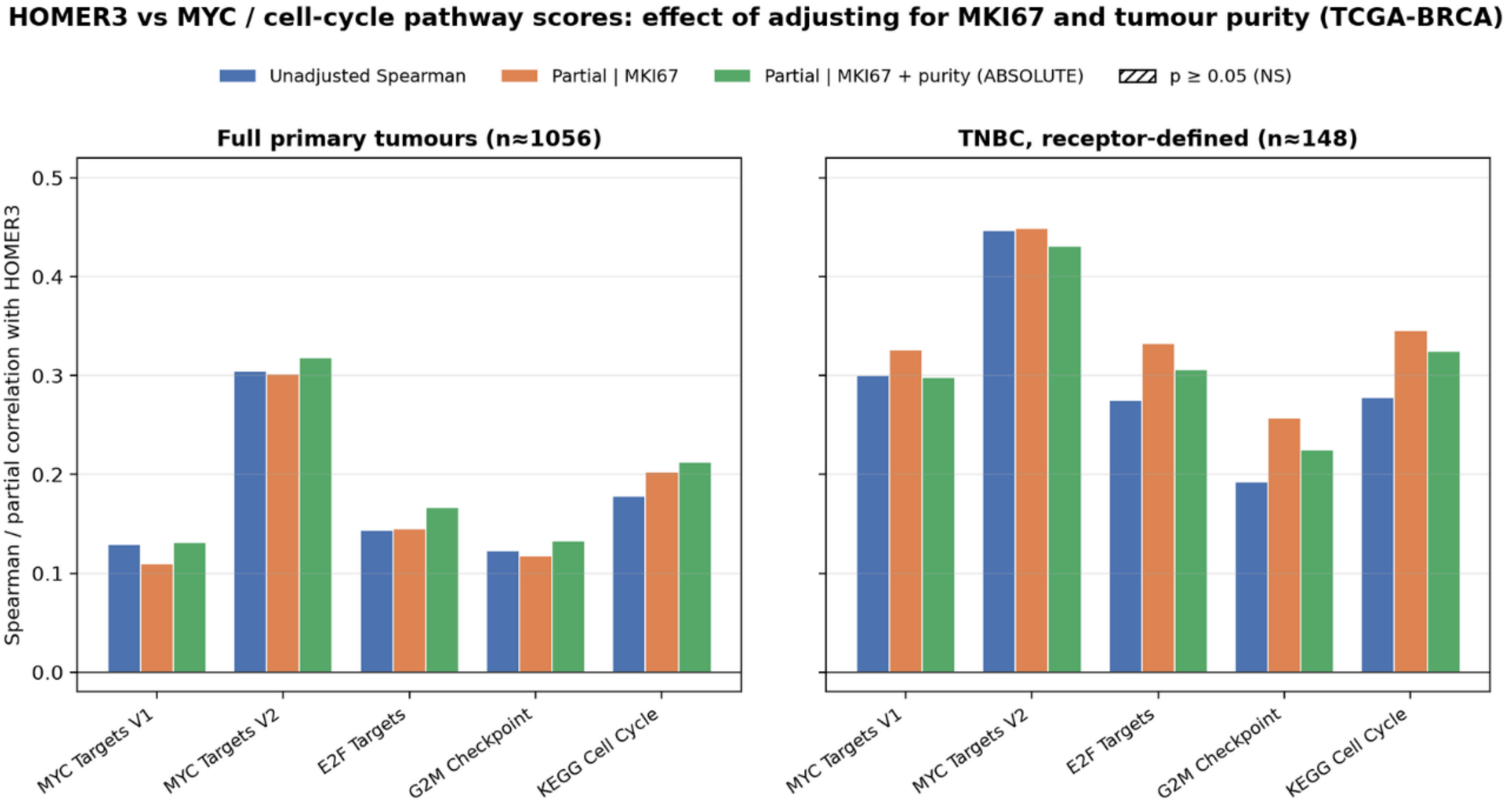

Figure S3. Correlation and partial correlation analyses between HOMER3 expression and MYC/cell-cycle pathway scores in TCGA-BRCA primary tumors. Bar plots show unadjusted Spearman correlations and partial correlations adjusted for MKI67 alone or MKI67 plus ABSOLUTE-estimated tumor purity in the full BRCA cohort and receptor-defined TNBC subset. HOMER3–pathway correlations were largely retained and remained statistically significant after adjustment, suggesting that these associations are not solely attributable to generalized proliferative activity or tumor purity.
