# Supplementary material for: Homer3 Promotes Aggressive Phenotypes in Triple-Negative Breast Cancer Through Cell Cycle- and MYC-Associated Programs
Source: Int J Mol Sci. 2026 Aug 4;27(15):7009. doi: 10.3390/ijms27157009 (PMC13467463; doi:10.3390/ijms27157009)
Supplement: Supplementary file 1 [file ijms-27-07009-s001.zip › revised_Figure S5.pdf]

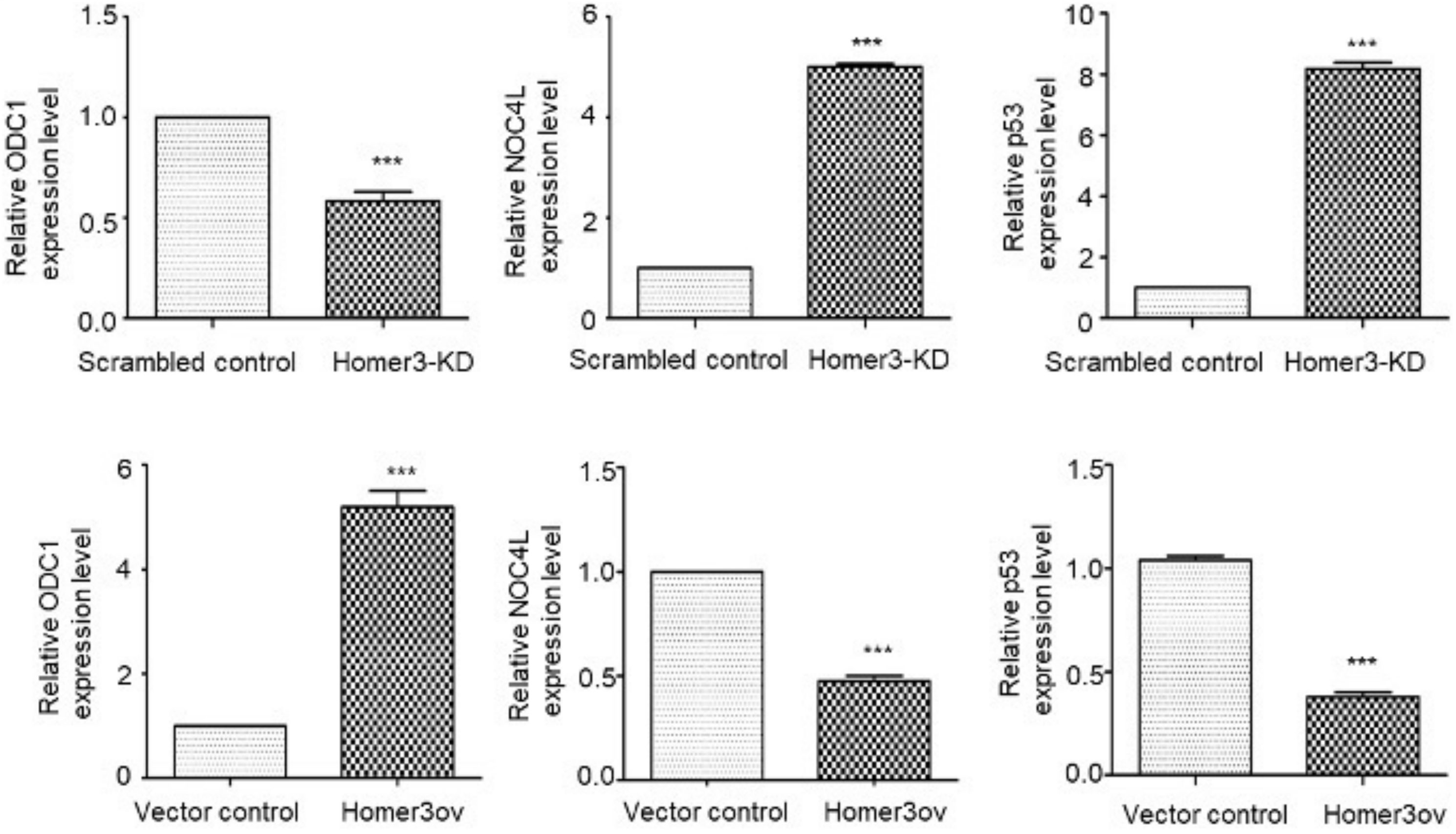

Figure S5. Homer3 knockdown decreased ODC1 but increased NOC4L and p53 expression, while Homer3 overexpression showed the opposite effects. It is worth noting that the NOC4L expression showed the opposite results in this qRT-PCR analysis compared to its TCGA analysis in Figure 7E.
