# Supplementary material for: Homer3 Promotes Aggressive Phenotypes in Triple-Negative Breast Cancer Through Cell Cycle- and MYC-Associated Programs
Source: Int J Mol Sci. 2026 Aug 4;27(15):7009. doi: 10.3390/ijms27157009 (PMC13467463; doi:10.3390/ijms27157009)
Supplement: Supplementary file 1 [file ijms-27-07009-s001.zip › revised_Table S1.pdf]

Table S1. Cox regression analyses of Homer3 expression and clinicopathological factors for overall survival in the TCGA-BRCA cohort.

Characteristics	Total(N)	Univariate analysis		Multivariate analysis	
		Hazard ratio (95% CI)	P value	Hazard ratio (95% CI)	P value
HOMER3	1,086				
Low	542	Reference			
High	544	1.209 (0.879 - 1.663)	0.244		
Age	1,086				
<= 60	603	Reference		Reference	
> 60	483	2.024 (1.468 - 2.790)	**< 0.001**	2.839 (1.795 - 4.491)	**< 0.001**
Pathologic stage	1,062				
Stage I&Stage II	800	Reference		Reference	
Stage III&Stage IV	262	2.367 (1.686 - 3.321)	**< 0.001**	3.219 (2.005 - 5.168)	**< 0.001**
ER status	1,036				
Positive	796	Reference		Reference	
Negative	240	1.410 (0.981 - 2.026)	0.063	1.835 (0.693 - 4.860)	0.222
PR status	1,033				
Positive	691	Reference		Reference	
Negative	342	1.372 (0.981 - 1.920)	0.065	1.223 (0.580 - 2.578)	0.596
HER2 status	717				
Positive	157	Reference		Reference	
Negative	560	0.628 (0.383 - 1.028)	0.064	0.921 (0.498 - 1.704)	0.793
PAM50	1,086				
Normal	40	Reference		Reference	
LumA	563	0.659 (0.302 - 1.438)	0.295	2.938 (0.383 - 22.568)	0.300
LumB	206	1.101 (0.486 - 2.495)	0.818	4.072 (0.516 - 32.138)	0.183
Her2	82	1.498 (0.621 - 3.614)	0.369	3.530 (0.419 - 29.746)	0.246
Basal	195	0.847 (0.371 - 1.935)	0.694	2.603 (0.330 - 20.512)	0.364
